# Evaluation of the relationship between intercostal muscle oxygenation measured by near-infrared spectroscopy and exercise capacity in group E COPD patients

**DOI:** 10.1007/s00424-024-02993-2

**Published:** 2024-07-24

**Authors:** Buğra Kerget, Gizem Çil, Alperen Aksakal

**Affiliations:** Department of Pulmonary Diseases, Ataturk University School of Medicine, 25240 Yakutiye, Erzurum Turkey

**Keywords:** COPD, Near-infrared spectroscopy, 6MWT

## Abstract

Near-infrared spectroscopy (NIRS) can be used to demonstrate muscle metabolism and oxygenation. NIRS-based oximeters enable the noninvasive measurement of static and dynamic muscle oxygenation. This study aimed to evaluate the relationship between NIRS readings and exercise capacity in group E COPD patients. The prospective study included 40 patients with group E COPD who presented to our outpatient clinic between May 2021 and June 2022. The patients were evaluated with pulmonary function testing, 6-Minute Walk Test (6MWT), echocardiography, and dyspnea and quality of life assessments. NIRS muscle oxygen saturation (SmO_2_) levels at the start and end of the 6MWT were obtained. 6MWT distance was positively correlated with intercostal SmO_2_ and fingertip SO_2_ at the start (*R* = 0.679, *p* ≤ 0.001 and *R* = 0.321, *p* = 0.04, respectively) and end of the 6MWT (*R* = 0.693, *p* ≤ 0.001 and *R* = 0.635, *p* ≤ 0.001, respectively) and negatively correlated with the number of hospitalizations due to exacerbations in the last year and mean pulmonary arterial pressure (*R* =  − 0.648, *p* ≤ 0.001 and *R* =  − 0.676, *p* ≤ 0.001, respectively). SF-36 score was positively correlated with intercostal SmO_2_ at the beginning of the 6MWT (*R* = 0.336, *p* = 0.03). Intercostal SmO_2_ levels at the start of the 6MWT positively correlated with diffusing capacity of the lung for carbon dioxide (DLCO) (*R* = 0.388, *p* = 0.01) and ratio of DLCO to alveolar volume (DLCO/VA) levels (*R* = 0.379, *p* = 0.02), and these correlations persisted more strongly after the 6MWT (*R* = 0.524, *p* = 0.01; *R* = 0.500, *p* = 0.01, respectively). NIRS is a practical and noninvasive method for measuring muscle oxygenation and can be used as an alternative to 6MWT in the evaluation of exercise capacity in patients with group E COPD.

## Introduction

Chronic obstructive pulmonary disease (COPD) presents with progressive airway obstruction despite appropriate medical therapies. Static hyperinflation, which is more pronounced in the emphysema phenotype, is later followed by dynamic hyperinflation, which leads to an increase in dead space volume. This can reach maximum levels in acute exacerbations of COPD [25].

Increasing hyperinflation initially manifests with dyspnea in COPD patients, followed by a reduction in exercise capacity [20]. Exercise intolerance is an important stage in the natural history of COPD and has a major impact on health-related quality of life, hospitalization rate, and survival [19, 20]. The 6-Minute Walk Test (6MWT) has gained importance in evaluating the functional exercise capacity of patients with COPD and other lung diseases such as pulmonary hypertension, interstitial lung diseases, and cystic fibrosis [10]. The 6MWT is a submaximal test but correlates well with cardiopulmonary exercise testing. It is simple, based on measuring the distance walked on a level surface in 6 min, and is well tolerated by patients [15]. The test is especially important in assessing the functional status of patients with severe COPD [11].

Although the 6MWT has an important place in functional assessment and is associated with patient effort, it is not always applicable for patients with severe and very severe COPD. As a result, various clinical and laboratory parameters and dyspnea scores have been tested to predict 6MWT performance [12, 15, 28]. Muscle oximetry based on near-infrared spectroscopy (NIRS) can provide noninvasive information about changes in muscle tissue oxygenation and hemodynamics based on the oxygen-dependent properties of near-infrared light. NIRS studies in COPD patients have shown that pulmonary rehabilitation has a favorable effect on both 6MWT performance and muscle oxygenation measured from the vastus lateralis [24]. In another study, it was observed that muscle oxygenation measured from different regions of the vastus lateralis during exercise showed heterogeneity in COPD patients [14]. A comparison of exercise and perfusion values of the quadriceps and intercostal muscles in COPD patients showed that the perfusion of the intercostal muscles was not preserved during exercise like the peripheral extremities [26].

NIRS studies have used the vastus lateralis muscle because of the ease of measurement, but we believe that oxygenation of the intercostal muscles during exercise is a more valuable indicator in COPD patients. In this study, we aimed to examine the correlation between intercostal muscle oxygenation measured by NIRS and 6MWT distance and other parameters in patients with severe COPD.

## Materials and methods

### Study design

This prospective study included 40 patients who had group E COPD according to the 2023 GOLD (Global Initiative for Chronic Obstructive Pulmonary Disease) guidelines and were followed up in the chest diseases outpatient clinic of our hospital [25]. Of the patients included in the study, 20 were male and 20 were female. The study was designed and conducted in accordance with the ethical principles set forth in the Declaration of Helsinki, and local ethics committee approval was obtained (B.30.2.ATA.0.01.00/427). All participants provided written informed consent. The study was supported by the Research Fund of Ataturk University (Project Number, 11581).

### Participants

Our study included patients aged 40 years and older with severe and very severe COPD who presented to the outpatient clinic of our hospital for follow-up between May 2021 and June 2022. Post-bronchodilator pulmonary function tests (PFT) were repeated to confirm the COPD diagnosis. An FEV_1_/FVC ratio < 70% and FEV_1_ < 50% predicted were sought as per the GOLD classification. The number of hospital admissions and/or hospitalizations due to acute exacerbation of COPD in the last year was evaluated according to the 2023 GOLD guideline. The patients’ records were examined retrospectively from the automation system of our hospital or the national online health data system (e-Nabiz). Patients with two or more acute exacerbations or at least one hospitalization were classified as group E.

Patients with complaints of increased dyspnea, cough, and sputum because of infection in the last 14 days, air pollution, or other triggering factors, as well as signs of tachypnea and/or tachycardia were evaluated for acute exacerbation of COPD and excluded from the study. Patients not meeting these criteria who were stable for the last 4 weeks and had fingertip saturation > 85% in room air were included in the study.

The primary outcome we targeted in our study shows the relationship between intercostal muscle oxygenation and exercise capacity in group E COPD patients. Secondary outcome is to show the relationship between intercostal muscle oxygenation and PFT, echocardiography, the number of COPD acute exacerbations, and the number of hospitalizations.

### Exclusion criteria

Patients with any potential contraindications to PFT (recent myocardial infarction, pulmonary embolism, cerebral aneurysm, active hemoptysis, pneumothorax, nausea, vomiting, and recent thoracic, abdominal, or ocular surgery) were identified before testing and excluded. Patients were also excluded from the study if they had any contraindications to the 6MWT, including second- or third-degree heart block, rapid ventricular or atrial arrhythmia, orthopedic disability, severe aortic stenosis, congestive heart failure, uncontrolled hypertension, debilitating neurological disease, dissecting aortic aneurysm, severe pulmonary hypertension, thrombophlebitis or intracardiac thrombus, acute pericarditis, PaCO_2_ > 70 mmHg in room air, or PaO_2_ < 40 mmHg in room air. In addition, mentally disabled or uncooperative patients were also excluded.

### PFT procedure

All tests were performed by the same technician who wore personal protective equipment to prevent transmission of infectious pathogens. The patients’ age, height, and weight were measured and recorded. The participants were asked to wear light, nonrestrictive clothing on the day of the test and were instructed to avoid smoking for 24 h, alcohol for 4 h, heavy meals for 2 h, and strenuous exercise for 30 min before testing. BTPS correction was performed according to room air and barometric pressure. The technician explained the expected maneuver to the patients and obtained three acceptable spirograms using a Cosmed Q-Box Body Plethysmography device. Those that met the 2019 ATS/ERS reproducibility and acceptability criteria for PFTs were included in the study [9]. The lower limits of the normal range determined for the healthy population were also calculated and presented by the spirometry device according to the criteria specified in the same report.

### Echocardiography

Echocardiographic examinations were performed by the same cardiologist using a Toshiba S270-A device with a 2.5-mHz probe before performing PFT and 6MWT. Examinations were performed in a quiet environment, with the patient in left recumbent position, calm, and breathing comfortably. All patients underwent cardiac auscultation, electrocardiography, and teleradiography before echocardiography. During the echocardiographic examination, parasternal long axis images, aorto-mitral-papillary muscle and parasternal short axis images from the apical level, and apical four-chamber and two-chamber images were obtained using two-dimensional, M-mode, color Doppler, pulse, and continuous Doppler modes, respectively. When necessary, the interarterial septum was also evaluated on subcostal echocardiographic images and the aortic arch on suprasternal echocardiographic images. Mean pulmonary arterial pressure (mPAP) values were determined based on tricuspid regurgitation recorded on echocardiography.

### 6MWT and fingertip saturation measurement

Before the 6MWT, the patients were asked to rest for at least 15 min and their oxygen saturation was measured using a fingertip pulse oximeter. Under physician supervision, the patients were instructed to walk as fast as they could on a 30-m course in a level corridor for 6 min. The test was terminated early in the event of any symptoms such as excessive fatigue, dyspnea, or palpitations to avoid endangering the patient. At the end of the test, the patient rested while the distance walked was recorded in meters. Borg dyspnea scores (0 for none and 10 for very severe) were obtained before and after the test [5].

### Near-infrared spectroscopy (MOXY® monitor) procedure

**Fig. 1:**
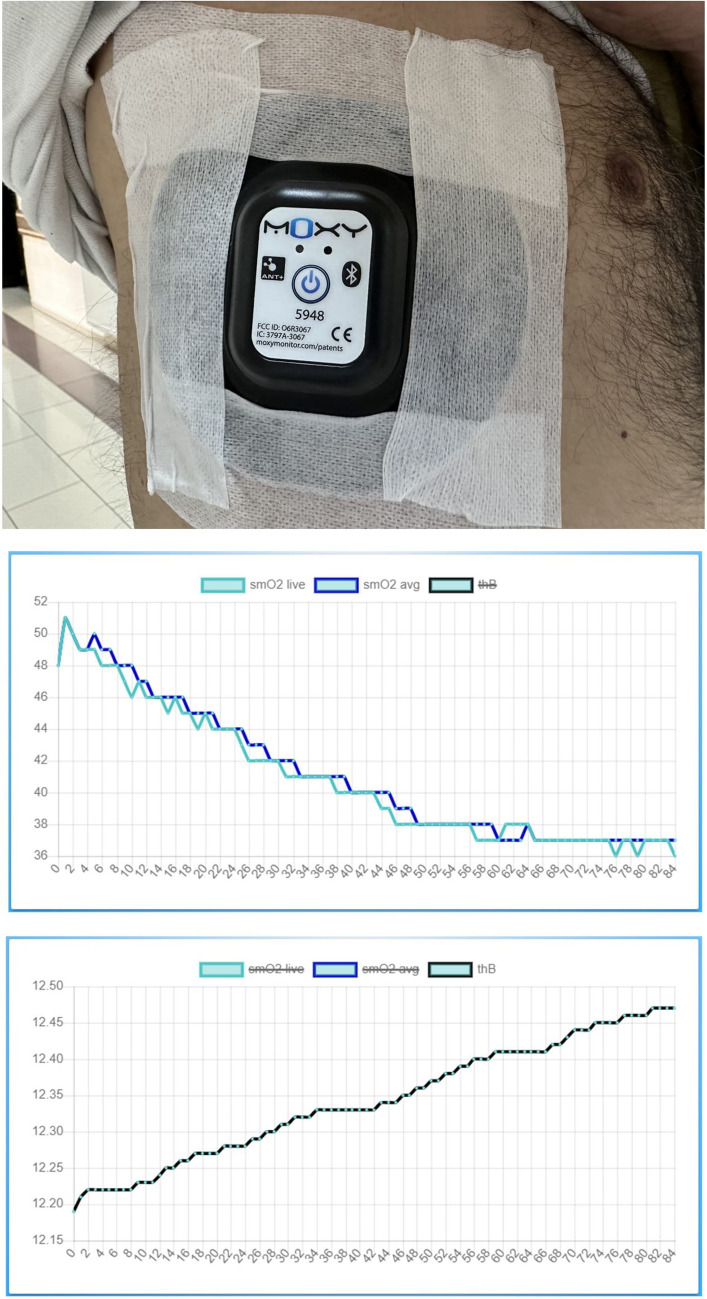
Placement of the near-infrared spectroscopy device (MOXY®) at the 5th intercostal space for intercostal muscle oxygenation (SmO_2_) measurement, example of the graphic display of muscle oxygen saturation (SmO2) and total hemoglobin (tHb) levels obtained using the MOXY monitor

Muscle oxygenation was measured by NIRS (MOXY, MN, USA). The sensor was placed over the right fifth intercostal space 2 cm anterior from its intersection with the anterior axillary line (Fig. 1). The device recorded for the entire duration of the 6MWT. MOXY measures the ratio of oxyhemoglobin concentration to total hemoglobin concentration in the muscle in real time and reports this as a percentage shown as muscle oxygen saturation or muscle oxygenation (SmO_2_). The collected data was stored in the internal memory of the device as a.csv file and was transferred to a Microsoft Excel file via Bluetooth connection between the device and a computer (Fig. 1). Intercostal muscle oxygenation levels measured in the first second at the beginning and the last second at the end of the 6MWT test were statistically analyzed.

## Statistical analysis

Before the study, G*Power analysis was used to determine the sample size for the study In calculating the sample size, the mean, standard deviation, and relationship levels of the groups obtained from previous studies or the ratios of the variables to each other should be known. Based on the hypotheses of this research, the study titled “The use of near-infrared spectroscopy for the evaluation of a 4-week rehabilitation program in patients with COPD” was examined [24]. In light of this information, *α* = 0.05 was taken as 1 – *β* = 0.9, and the sample size was calculated with an effect size of 0.5714286. According to the result, it was decided to include a minimum of 40 patients. Analyses were performed using IBM SPSS Statistics version 20.0 software (IBM Corp, Armonk, NY). Pearson’s chi-square test and Mann–Whitney *U* test were used for between-group comparisons of normally and nonnormally distributed numerical data, respectively. The Wilcoxon test was used to compare normally distributed parameters at the beginning and end of the 6MWT. Multivariate logistic regression analysis was performed to evaluate the effect of multiple parameters on 6MWT distance. Relationships between two quantitative variables were examined using Pearson’s correlation analysis if normally distributed and Spearman’s correlation analysis if nonnormally distributed. *p* values < 0.05 were considered statistically significant.

## Results

The mean age of the patients was 60.5 ± 11.1 years. The average age of female patients included in our study was 62.5 ± 9.4 years, while the average age of male patients was 61.6 ± 10.2 years. In the statistical analysis of the patients according to gender, no significant difference was observed between the average ages (*p* = 0.56). Comorbidities included hypertension in 23 patients (57.5%), diabetes mellitus in 10 patients (25%), and previous coronary artery disease in 6 patients (15%). All patients were ex-smokers and had a smoking history of 61.5 ± 40.5 pack-years.

The patients’ demographic data, PFT results, number of acute exacerbations and hospitalizations due to exacerbation in the last year, and echocardiographic findings are shown in Table 1. The patients’ mean 6MWT distance was 345.4 ± 101.4 m. During the 6MWT, intercostal SmO_2_ and fingertip SO_2_ levels decreased statistically compared to baseline (*p* < 0.001 for both) while fatigue, dyspnea, and total hemoglobin levels increased (*p* = 0.001, 0.002, < 0.001, respectively). Age and BMI were both inversely correlated with SmO_2_ level at the start of the 6MWT (*R* =  − 0.516, *p* = 0.01 and *R* =  − 0.570, *p* = 0.01, respectively).

**Table 1:** Characteristics of the group E COPD patients included in the study

	Group E COPD patients (*n* = 40) Mean ± SD
Age (years)	60.5 ± 11.1
Smoking history (pack-years)	61.5 ± 40.5
BMI	26.4 ± 4.2
FEV_1_ (L)	1.2 ± 0.5
FEV_1_ (%)	43.7 ± 17.5
FVC (L)	2.7 ± 0.8
FVC (%)	73 ± 18.6
TLC (%)	133.2 ± 34.9
RV (%)	251.1 ± 97.4
RV/TLC	1.8 ± 0.3
DLCO (%)	74.1 ± 14.2
DLCO/VA (%)	87 ± 11.8
COPD exacerbations in the last year	7.7 ± 7.5
Hospitalizations for COPD exacerbations in the last year	1.9 ± 1.3
EF (%)	54.3 ± 2.9
Mean PAP (mmHg)	42.5 ± 16.2

*COPD*, chronic obstructive pulmonary disease; *BMI*, body mass index; *FVC*, forced vital capacity; *FEV*_*1*_, forced expiratory volume in 1 s; *TLC*, total lung capacity; *RV*, residual volume; *DLCO*, diffusing capacity of the lung for carbon monoxide; *DLCO/VA*, ratio of DLCO to alveolar volume; *mMRC*, modified Medical Research Council scale; *CAT*, COPD Assessment Test; *EF*, ejection fraction; *PAP*, pulmonary arterial pressure

The results of correlation analysis between clinical, PFT, 6MWT, and echocardiographic parameters are shown in Tables 2 and 3. Intercostal SmO_2_ levels at the start of the 6MWT positively correlated with diffusing capacity of the lung for carbon dioxide (DLCO) (*R* = 0.388, *p* = 0.01) and DLCO/alveolar volume ratio (*R* = 0.379, *p* = 0.02), and these correlations persisted more strongly after the 6MWT (*R* = 0.524, *p* = 0.001 and *R* = 0.500, *p* = 0.001, respectively). DLCO also showed a positive correlation with 6MWT distance (*R* = 0.737, *p* ≤ 0.001). 6MWT distance was positively correlated with intercostal SmO_2_ and fingertip SO_2_ at the start of the 6MWT (*R* = 0.679, *p* ≤ 0.001 and *R* = 0.693, *p* ≤ 0.001, respectively) and the end of the 6MWT (*R* = 0.321, *p* = 0.04 and *R* = 0.635, *p* ≤ 0.001, respectively) and negatively correlated with the number of hospitalizations due to exacerbations in the last year (*R* =  − 0.648, *p* ≤ 0.001) and mean pulmonary arterial pressure (PAP) (*R* =  − 0.676, *p* ≤ 0.001).

**Table 2:** Correlation analysis of patients’ pulmonary function parameters, 6MWT distance, and intercostal SmO_2_ levels at the start and end of the 6MWT

		FEV_1_ (%)	FVC (%)	FEV_1_/FVC	TLC (%)	RV (%)	RV/TLC	DLCO (%)	DLCO/VA	SmO_2_ at start of 6MWT	SmO_2_ at end of 6MWT	6MWT distance (m)
FVC (%)	*R*	0.707**	1									
	*p*	< 0.001										
FEV_1_/FVC	*R*	0.830**	0.347*	1								
	*p*	< 0.001	0.028									
TLC (%)	*R*	− 0.226	− 0.048	− 0.223	1							
	*p*	0.160	0.770	0.166								
RV (%)	*R*	− 0.404**	− 0.270	− 0.372	0.920**	1						
	*p*	0.010	0.092	0.018	< 0.001							
RV/TLC	*R*	− 0.581**	− 0.539**	− 0.538**	0.534**	0.813**	1					
	*p*	< 0.001	< 0.001	< 0.001	< 0.001	< 0.001						
DLCO (%)	*R*	0.303	0.334*	0.159	− 0.210	− 0.439**	− 0.584**	1				
	*p*	0.057	0.035	0.326	0.194	0.005	< 0.001					
DLCO/VA	*R*	0.463**	0.322*	0.312*	0.124	− 0.044	− 0.283	0.467**	1			
	*p*	0.003	0.043	0.050	0.445	0.787	0.076	0.002				
SmO_2_ at start of 6MWT	*R*	0.112	0.160	0.053	0.251	0.127	− 0.093	0.388*	0.379*	1		
	*p*	0.492	0.323	0.744	0.118	0.435	0.568	0.01	0.02			
SmO_2_ at end of 6MWT	*R*	0.246	0.220	0.122	0.008	− 0.078	− 0.193	0.524**	0.500**	0.760**	1	
	*p*	0.126	0.173	0.453	0.959	0.631	0.232	0.001	0.001	< 0.001		
6MWT distance (m)	*R*	0.251	0.308	0.184	− 0.034	− 0.240	− 0.422**	0.737**	0.323*	0.679**	0.693**	1
	*p*	0.118	0.053	0.255	0.835	0.136	0.007	< 0.001	0.042	< 0.001	< 0.001	

*FVC*, forced vital capacity; *FEV*_*1*_, forced expiratory volume in 1 s; *TLC*, total lung capacity; *RV*, residual volume; *DLCO*, diffusing capacity of the lung for carbon monoxide; *DLCO/VA*, ratio of DLCO to alveolar volume; *SmO*_*2*_, muscle oxygen saturation; *6MWT*, 6-Minute Walk Test

^*^Correlation is significant at the < 0.05 level (2-tailed). **Correlation is significant at the < 0.01 level (2-tailed)

**Table 3:** Correlation analysis of fingertip SO_2_ and intercostal SmO_2_ before and after the 6MWT, numbers of COPD exacerbations and hospitalizations in the last year, and echocardiographic findings

		6MWT distance (m)	SmO_2_ at start of 6MWT	SmO_2_ at end of 6MWT	SO_2_ at start of 6MWT	SO_2_ at end of 6MWT	Number of COPD exacerbations in the last year	Number of hospitalizations for COPD exacerbations in the last year	EF (%)	Mean PAP (mmHg)
SO_2_ at start of 6MWT	*R*	0.321*	0.078	0.337*	1					
	*p*	0.04	0.632	0.034						
SO_2_ at end of 6MWT	*R*	0.635**	0.261	0.636**	0.719**	1				
	*p*	< 0.001	0.104	< 0.001	< 0.001					
Number of COPD exacerbations in the last year	*R*	− 0.178	− 0.004	− 0.197	− 0.048	− 0.228	1			
	*p*	0.271	0.981	0.224	0.768	0.156				
Number of hospitalizations for COPD exacerbations in the last year	*R*	− 0.648**	− 0.233	− 0.210	0.037	− 0.450**	0.358*	1		
	*p*	< 0.001	0.149	0.194	0.819	0.004	0.023			
EF (%)	*R*	0.232	0.349*	0.164	0.113	− 0.106	0.167	0.194	1	
	*p*	0.150	0.027	0.311	0.486	0.513	0.303	0.230		
Mean PAP (mmHg)	*R*	− 0.676**	− 0.375*	− 0.693**	− 0.287	− 0.732**	0.240	0.493**	0.134	1
	*p*	< 0.001	0.017	< 0.001	0.073	< 0.001	0.136	0.001	0.410	

*COPD*, chronic obstructive pulmonary disease; *6MWT*, 6-Minute Walk Test; *SmO*_*2*_, muscle oxygen saturation; *SO*_*2*_, fingertip oxygen saturation in room air; *EF*, ejection fraction; *PAP*, pulmonary arterial pressure

^*^Correlation is significant at the < 0.05 level (2-tailed). **Correlation is significant at the < 0.01 level (2-tailed)

The multivariate logistic regression analysis of the association between 6MWT distance and age, BMI, intercostal SmO_2_ and fingertip SO_2_ at the start of the 6MWT, the numbers of COPD exacerbations and related hospitalizations in the last year, and mean PAP is shown in Table 4. 6MWT distance was positively associated with higher SmO_2_ and SO_2_ levels at the start of the 6MWT (*p* = 0.003 and < 0.001, respectively) and negatively associated with the number of hospitalizations due to COPD exacerbations in the last year and mean PAP (*p* < 0.001 and 0.006, respectively).

**Table 4:** Multivariate logistic regression analysis of the effect of age, BMI, intercostal SmO_2_ and fingertip SO_2_ at the start of the 6MWT, numbers of COPD exacerbations and hospitalizations in the last year, and mean PAP on 6MWT distance

	Unstandardized coefficients		Standardized coefficients	*t*	*p*	95% confidence interval for *B*	
	*B*	Std. error	Beta			Lower bound	Upper bound
(Constant)	− 93.157	178.031		− 0.523	0.604	− 455.795	269.481
Age (years)	− 0.572	0.737	− 0.063	− 0.776	0.443	− 2.072	0.929
BMI (kg/m^2^)	− 5.249	2.142	− 0.220	− 2.451	0.020	− 9.611	− 0.886
SmO_2_ at start of 6MWT	1.851	0.616	0.291	3.007	0.003	0.597	3.105
SO_2_ at start of 6MWT	7.492	1.874	0.285	3.998	0.000	3.675	11.309
Number of COPD exacerbations in the last year	0.818	0.985	0.060	0.831	0.412	− 1.188	2.825
Number of hospitalizations for COPD exacerbations in the last year	− 37.756	6.274	− 0.484	− 6.018	0.000	− 50.536	− 24.977
Mean PAP (mmHg)	− 1.605	0.542	− 0.256	− 2.961	0.006	− 2.710	− 0.501

*BMI*, body mass index; *SmO*_*2*_, muscle oxygen saturation; *SO*_*2*_, fingertip oxygen saturation in room air; *6MWT*, 6-Minute Walk Test; *EF*, ejection fraction; *PAP*, pulmonary arterial pressure

## Discussion

This study showed that of the PFT parameters, DLCO was a better marker of both 6MWT distance and resting intercostal SmO_2_ level. 6MWT distance was also positively correlated with resting intercostal SmO_2_ level and negatively correlated with the number of hospitalizations in the last year and mean PAP. Of the dyspnea and quality of life measures used, SF-36 best correlated with resting intercostal SmO_2_ levels. The results of multivariate regression analysis showed that fingertip saturation in room air and resting intercostal muscle oxygenation were significant predictors of 6MWT distance.

Increased respiratory work of breathing in the advanced stages of COPD causes a significant increase in the workload of both the inspiratory and expiratory auxiliary respiratory muscles. Improving perfusion is attempted in order to reduce the work of breathing and oxygen demand in hypoxic patients [7, 19, 22]. Increased need for ventilation causes perfusion to be concentrated in the auxiliary respiratory muscles rather than the peripheral extremities. During exercise, restricted intercostal muscle blood flow, increased quadriceps blood flow, and a plateau in cardiac output are signs that the cardiovascular system is unable to meet the energy demand of the muscles [26]. This may lead to activity limitations, atrophy of the extremities, and atelectasis associated with inability to eliminate secretions due to movement limitation.

The 6MWT continues to be used as the main tool for demonstrating individuals’ activity levels. This test provides clinicians insight into patients’ levels of participation in activities of daily living and instrumental activities of daily living [5]. Therefore, the 6MWT is frequently used by both pulmonologists and physiotherapists as a concrete measure reflecting the effectiveness of treatment [3]. A 6MWT test distance below 350 m in COPD patients has also been associated with high mortality [16, 27]. In severe COPD patients, the progressive disease course may progress with cardiovascular complications starting with pulmonary hypertension and followed by myocardial infarction, ventricular or atrial arrhythmias, and uncontrolled hypertension [6]. Hypoxia during exercise may also impair cardiac perfusion during testing and lead to complications [8]. This led to the investigation of alternative scoring systems to enable the measurement of exercise capacity in patients with severe COPD.

CAT and mMRC scores are used to assess patients’ dyspnea and quality of life. GOLD recommends using one of these tests to grade disease severity [25]. GOLD also recommends modifying COPD patients’ pharmacological treatment based on the results of these questionnaires [18, 25]. Studies have shown that both scores can be effective in predicting 6MWT distance [28]. In addition, SF-36 scoring has been used to evaluate quality of life [23]. SF-36 can provide important insight regarding the effectiveness of pharmacological and nonpharmacological treatments in COPD patients [4]. Studies have also shown that SF-36 score is negatively correlated with mMRC and post-6MWT Borg dyspnea score and positively correlated with 6MWT distance [23].

In our study, we observed that among the PFT parameters, DLCO was positively correlated with both 6MWT distance and intercostal SmO_2_ level at rest. In spite of increased lung volume in COPD patients, the concomitant increase in alveolar wall and capillary destruction result in a loss of diffusion capacity [1]. This progresses with severe COPD and is the main cause of desaturation during effort and activity limitation in these patients. The fact that FEV_1_, which is used as an indicator of the degree of obstruction according to the GOLD classification, did not correlate with the parameters examined in our study suggests that FEV_1_ should not be used routinely to evaluate participation in activities of daily living in individuals in COPD. When at rest, intercostal SmO_2_ level was better correlated with 6MWT distance compared to fingertip SO_2_. Hospitalizations due to acute exacerbations of COPD were inversely correlated with mean PAP [17]. The auxiliary respiratory muscles are used more in COPD because of the increased work of breathing [13]. Previous studies in the literature have mostly focused on perfusion of the peripheral extremities in COPD [2, 14, 21]. With movement, increased oxygen demand in the peripheral extremities enhances circulation to this area. However, the results of our study show that even resting intercostal SmO_2_ level is an important factor in effort capacity. Intercostal SmO_2_ decreased further with movement and the value obtained at the end of the 6MWT was a better indicator of 6MWT distance than the resting measurement. In the later stages of COPD, frequent episodes of recurrent hypoxia lead to elevated mean PAP and effort limitation in patients.

The correlation between 6MWT distance and our findings can be evaluated accordingly. Multivariate logistic regression analysis indicated that intercostal SmO_2_ level was associated with 6MWT distance but to a lesser extent than fingertip SO_2_ level. We attributed this to the fact that intercostal SmO_2_ level was more affected by age and BMI than SO_2_ level in the analysis. In the correlation analysis of 6MWT distance and resting intercostal SmO_2_ levels with dyspnea and quality of life measures, the SF-36 questionnaire was better correlated with intercostal SmO_2_ level. This may be related to the more detailed analysis in the SF-36 questionnaire compared to the other tests.

The fact that we had a single device for muscle oxygenation measurement in our study caused peripheral extremity evaluation not to be performed simultaneously. In addition, our study aimed to evaluate the relationship between intercostal muscle oxygenation and many parameters, especially PFT and 6MWT, in COPD patients. For this reason, COPD patients were evaluated among themselves. Still, studies in which the healthy control group and group A and B COPD patients were also assessed may be more guiding.

In conclusion, fingertip SO_2_ levels are used in outpatient clinic examinations of in COPD patients because it is quick and practical, but it was not as effective as intercostal SmO_2_ level in indicating patients’ effort capacity. The 6MWT cannot always be performed to assess effort capacity in patients with severe COPD. Therefore, intercostal SmO_2_ measurement is a noninvasive and easily performed test that may offer a new approach to evaluating patients’ walking distances.

## Data Availability

The data that support the findings of this study are available from the corresponding author upon reasonable request.
